# Give Us Proof

**Published:** 1885-12

**Authors:** 


### GIVE US PROOF.

In a letter to Dr. George B. Peck,* Dr. T. F. Allen, of New York, writes:

"If the efficacy of infinitesimals can be demonstrated on the healthy, and the provings therefrom utilized, then they will be, I believe, the best implements to use; for the less medicine prescribed to patients, the better the results. For nearly twenty years I prescribed almost exclusively the two-hun-

---

* See *Medical Advance* for October, 1885, p. 177.

dredth potencies, but for the last few years I have been prescribing mainly the third and sixth attenuation, and have about come to the conclusion that my results, in most cases, are more prompt and far-reaching than formerly with the two-hundredths. I have always found that fewer doses of these lower attenuations can be prescribed, that whereas formerly I could repeat the two-hundredth in water with impunity every hour or two, now I get my best results from single doses, much better, indeed, than formerly from single doses of the high potency.

"My present status is that of a pure Hahnemannian, giving as a rule infrequent doses of the moderate attenuation, waging an unsparing warfare upon allopathic expedients of all sorts. Those who know me well will bear me out in saying that no one more faithfully studies the materia medica, more carefully prescribes the indicated remedy, and in every respect is a more consistent homœopathist than I am to-day. I have no tolerance for those who alternate their medicines and overdose their patients. I cannot tolerate those who have departed from the master's rules and use mainly fluxion potencies, very frequently on empirical indications. My motto is, 'Prove all things and hold fast that which is good.'

"I believe there has been no demonstration of dynamization and no proof of the power of infinitesimals, and I will not be an apostle of these dogmas until they have been proved to be God's truths. I have worked and fought for them and for the right of free speech in their favor and will still fight for it; but, since after years of honest work to prove their truth I have failed, I can do no less than boldly announce the fact and solicit the help of all who have the future of Homœopathy and of accurate therapeutics at heart."

The power of dynamized remedies must be shown by exact experiment, not by argument. Let us, then, briefly glance at the facts of the case. On the one side we find numerous educated physicians very successful in curing the sick, who have used both the low potencies and the high, declare in favor of the high.

*Secondly*, we have numerous provings—and these pathogenses have been verified clinically—made by high potencies. Records of these provings will be found in Dr. Allen's *Encyclopædia*. As opposed to this evidence we have the assertions of men who have used the low potencies only or chiefly that the high potencies are of no value.

Dr. Dunham wrote:* "Experience shows that while the

* See *Homœopathy, The Science of Therapeutics*, p. 256.

majority of cases, both acute and chronic, are cured more speedily by the high than by the lower potencies, yet, in some cases, the converse is observed."

We feel warranted in asserting that Drs. A. Lippe, Edward Bayard, P. P. Wells, David Wilson, etc., have been using high potencies these forty years and have always found them active and reliable.

Now, then, since Dr. Allen has tried both the high and the low potencies, and as his experience with them has been unique and uncommon, it would certainly be very welcome to his *confreres* to learn the facts upon which he bases his changed views—upon which he basis this opinion: "I believe there has been no demonstration of dynamization and no proof of the powers of infinitesimals." A sweeping assertion; prove it if possible!

We take the liberty of quoting below a few cases cured by Dr. Allen with high potencies. Will he kindly quote some from his case book showing where high potencies failed and low potencies (the third or sixth attenuation) cured? E. J. L.

### CASES ILLUSTRATING THE ACTION OF HIGH POTENCIES.

CASE I.—February 25th.—Boy aged two and a-half years. For two months has had sore eyes. Found it very difficult to examine his eyes on account of excessive photophobia. Profuse muco-purulent discharge, with red, swollen lids and face; congestion of bulb and lids; pustule on each cornea. *Euphrasia.*

March 1st.—Discharge all gone; opens his eyes himself and looks up; lids and face still red. *Sac. lac.*

March 19th.—Somewhat worse again. *Euphrasia.*

March 24th.—No improvement; afraid of examination; photophobia very great; the external canthi are cracked, sore, and bleed; eczematous eruptions on face about his eyes. *Graphites*$^{35m}$, one dose. After this his eyes steadily improved; eruptions came out more on his face and became quite annoying; continued *Sac. lac.*

April 15th.—Reported entirely well.—*T. F. Allen, M. D.* (*Journal of Mat. Med.*, 1868, *p.* 85).

CASE II.—Keratitis pustulosa. Girl eight years old. Has been subject to repeated attacks of pustular keratitis.

February 24th.—Pustule on cornea, with lachrymation and photophobia. (Noises in the ear disappear when the eyes become sore.) With this and every attack of sore eyes, the top of her head becomes so sore that she cannot comb her hair. *Merc. sol.*$^{2c}$, one dose.

February 25th.—Much better.

March 12th.—Has been well till yesterday, when a fresh pustule began to form; has had no noises in ears, and has no soreness on the head; lachrymation profuse; photophobia moderate; worse toward evening. Prescribed *Pulsatilla* 2C, one dose.

March 19th.—Pustule disappeared; slight inflammation still remains. *Sac. lac.*

April 1st.—No inflammation; slight macula of cornea. *Calc. carb.*$^{2c}$.

August 30th.—Nebula all gone. *Calc. carb.*$^{81m}$ one dose.—*T. F. Allen, M. D.* (*Journal of Mat. Med., p.* 75, 1869).

Case III.—September 15th.—Girl two and one-fourth years old. Ulcer of cornea. Small round ulcer in centre of cornea. *No photophobia.* Edges of lids thick and red; child pale, unhealthy, fat, and flabby; eats heartily. *Kali c.*$^{cc}$, one dose.

September 19th.—Better. Sleeps better at night.

September 27th.—Ulcer nearly healed.

November 8th.—Ulcer well. Lids remain sore and excoriated. *Graphites*$^{35m}$.

November 13th.—Bulb inflamed; the ulcer has broken out afresh and is very deep; slight circumcorneal redness; *one and a half lines of hypopyon in ant. chamber.* No vascularity of cornea, no photophobia, very restless nights. *Sulphur* $^{cc}$, one dose.

November 15th.—Healing rapidly. *Hypopyon entirely gone;* so, also, inflammation from conj.; eye still watery and suffused. —*T. F. Allen, M. D.* (*Journal Mat. Med., p.* 86, 1869).

Case IV.—A young woman had suffered a long time with a "kind of neuralgia." The head felt constantly as if it were a cushion, and some one were pressing their two fingers in it at the occiput as if feeling for pins, with occasional lightning-like flashes in the eyes and feeling as if something obscured vision. This condition in cold weather or in a draught; *relieved by wrapping the head up warmly. Silicea*$^{72m}$, one dose, speedily and permanently removed all trouble. She seemed otherwise well.—*T. F. Allen, M. D.* (*Journal of Mat. Med., p.* 61, 1870).

